# Supplementary material for: Sirtuin1 Mediates the Protective Effects of Echinacoside against Sepsis-Induced Acute Lung Injury via Regulating the NOX4-Nrf2 Axis
Source: Antioxidants (Basel). 2023 Oct 29;12(11):1925. doi: 10.3390/antiox12111925 (PMC10669561; doi:10.3390/antiox12111925)
Supplement: Supplementary file 1 [file antioxidants-12-01925-s001.zip › antioxidants-2632029-supplementary.pdf]

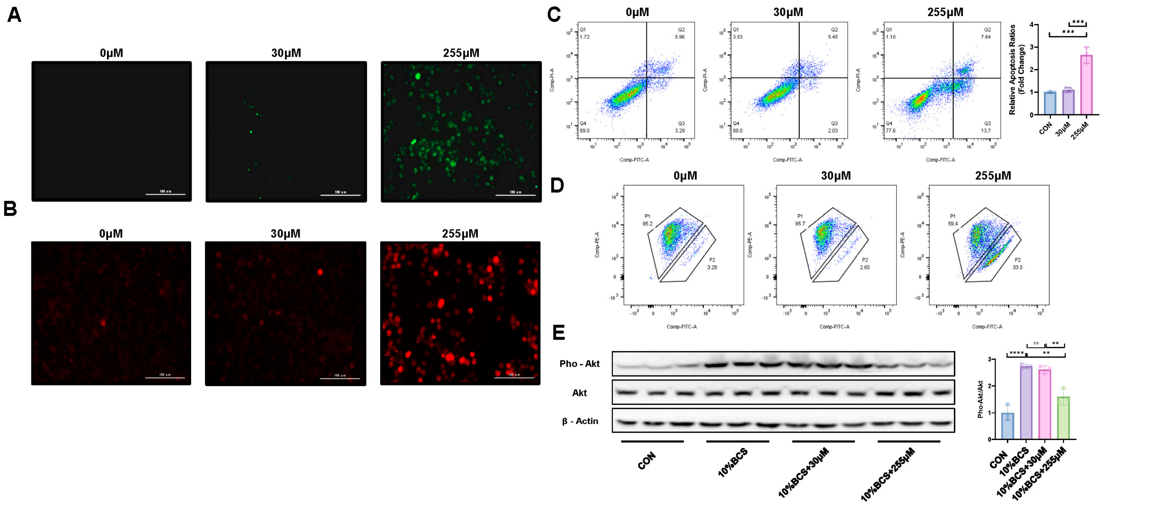

Supplementary Figure S1: The following supporting information can be downloaded at: www.mdpi.com/xxx/s1, Figure S1 : (A) Cells were incubated with H2DCFDA for 30 min to determine the ROS content. Bars represent 100 μm. (B) MitoROS in HUVECS by Mitosox red staining. Bars represent 100 μm. (C) The Annexin V-FITC Apoptosis Detection Kit was used to detect cell apoptosis, and the data were analyzed by FlowJo. (D) Flow cytometry based on JC-1 staining was conducted to evaluate mitochondrial function. (E) After subjecting the cells to starvation, they were observed for their effects on P-AKT and AKT under conditions of starvation (CON) and treatment with different drug concentrations (0 μM, 30 μM, and 255 μM) in the presence of 10% bovine calf serum (BCS). Data are presented as mean ± SD. (** $p < 0.01$, *** $p < 0.001$).
